# The smoke-free home study: study protocol for a cluster randomized controlled trial of a smoke-free home intervention in permanent supportive housing

**DOI:** 10.1186/s12889-022-14423-y

**Published:** 2022-11-14

**Authors:** Rachel Odes, Jessica Alway, Margot Kushel, Wendy Max, Maya Vijayaraghavan

**Affiliations:** 1 National Clinician Scholars Program, University of California San Francisco, San Francisco, CA USA; 2 Division of General Internal Medicine, University of California San Francisco, CA San Francisco, USA; 3 Division of Vulnerable Populations, University of California San Francisco, San Francisco, CA USA; 4 Institute for Health & Aging, School of Nursing, University of California San Francisco, San Francisco, CA USA

**Keywords:** Smoking, Tobacco, Homeless, Smoke-free home, Tobacco-cessation, Permanent supportive housing, Community-based

## Abstract

**Background:**

Formerly chronically homeless adults who live in permanent supportive housing (PSH) have high prevalence of smoking. It is uncommon to find smoke-free policies in PSH because of the concern that such policies contradict PSH’s harm reduction framework and could increase homelessness should residents lose their housing because of the policy. However, in the absence of such policies, non-smoking PSH residents face the harmful effects of secondhand smoke exposure while residents who smoke see increased risks from high rates of smoking throughout their residence. Our pilot work highlighted the feasibility and acceptability of an intervention designed to promote voluntary adoption of a smoke-free home. Here we report a protocol for a cluster randomized controlled trial of the smoke-free home intervention for formerly chronically homeless residents in PSH.

**Methods:**

The smoke-free home intervention provides face-to-face counseling and instruction to PSH residents on how to adopt a smoke-free home and offers training for PSH staff on how to refer residents to tobacco cessation services. We will randomize 20 PSH sites in the San Francisco Bay Area to either the intervention or wait-list control arms. We will enroll 400 PSH residents who smoke cigarettes in their housing unit and 120 PSH staff who work at the sites. At baseline, three- and six-months follow-up, we will ask residents to report their tobacco use and cessation behaviors and adoption of smoke-free homes. We will ask staff to answer questions on their knowledge, attitudes, practices, and barriers related to supporting residents’ smoking cessation. The primary outcome for PSH residents is adoption of smoke-free homes for 90 days or more at six-months follow-up, and the secondary outcome is point prevalence tobacco abstinence. The primary outcome for PSH staff is change in Smoking Knowledge Attitudes Practices survey score.

**Discussion:**

Voluntary adoption of smoke-free homes is a promising approach for reducing exposure to secondhand smoke and reducing tobacco use among a population facing high rates of tobacco-related disease, and is aligned with PSH’s harm reduction framework. Findings from this study have the potential to inform adoption of tobacco control policies among vulnerable populations most at risk for smoking-related harms.

**Trial registration:**

This study was registered with the U.S. National Institute of Health Clinical Trials register on April 22, 2021: NCT04855357.

## Background

Tobacco use is more prevalent among low-income and socially marginalized communities than their affluent counterparts, contributing to an unequal burden of tobacco-related diseases, a key health equity issue [1]. Previously chronically homeless individuals residing in permanent supportive housing (PSH) have borne the brunt of structural inequities leading to high rates of tobacco use [1, 2]. PSH is subsidized housing with closely linked or on-site voluntary medical and/or social services for individuals with a history of chronic homelessness and co-occurring mental health conditions, substance use disorders, and disabilities [3, 4]. Chronic homelessness is defined by the U.S. federal government as being continuously homeless for more than 1 year, or having four or more episodes of homelessness over 3 years, which combine to more than 1 year, in addition to having a disabling condition. Approximately 25% of people experiencing current homelessness in the U.S. meet these criteria for chronic homelessness [4]. PSH is an evidence-based approach to ending homelessness and represents the largest type of residential program in the U.S. for individuals who were previously homeless, with over 376,000 beds throughout the country [5]. Housing first is the most common and preferred approach to PSH. Rooted in harm reduction, housing first approaches have no preconditions of abstinence from alcohol or substances and do not require residents to engage in supportive services prior to obtaining or maintaining housing [3].

Negative social determinants of health are associated with high rates of tobacco use among people who experience homelessness [1]. These determinants include extreme poverty and the social marginalization that results from being homeless, increased availability of tobacco products in low-income communities, and limited access to cessation resources and smoke-free housing [1, 6]. Approximately 50% of those living in PSH report current smoking [7]. Mortality rates among PSH residents are at least double that of age-matched individuals in the general population [8] and tobacco-caused chronic diseases including cancers and cardiovascular disease contribute to over 60% of the all-cause mortality of PSH dwelling individuals [8]. Housing inequities disproportionately expose disadvantaged communities to secondhand smoke (SHS) and other cancer risks and contribute to poor access to preventive health care such as cancer screenings [9].

Tobacco use also increases financial strain on this already at-risk population. PSH residents in the San Francisco Bay Area who smoked spent 12% (median, range 5–26%) of their monthly income on tobacco [10]. PSH residents who have an income are required to pay up to 30% of their earnings on their monthly rent, and tobacco spending may interfere with residents’ ability to pay rent [11]. Tobacco expenditures may also interfere with residents’ ability to purchase food and increase risk for food insecurity [12]. Despite high rates of tobacco use and its detrimental impact on PSH residents’ health and financial well-being [12], PSH typically do not have mandated smoke-free policies restricting indoor smoking in living areas nor do they offer cessation services [11, 13].

### Tobacco control initiatives

Smoke-free policies and smoking cessation services are among the most effective tobacco control interventions. The Department of Housing and Urban Development (HUD) mandated a smoke-free policy that restricted indoor smoking in public housing authority housing, effective July 2018, and in some housing authority sites, that mandate is accompanied with access to cessation resources [14, 15]. In contrast, smoke-free policies are uncommon in PSH. There is a concern that mandated smoke-free policies may conflict with PSH’s harm-reduction framework, potentially increasing risk of evictions [11]. PSH staff may lack resources and time to enforce a smoke-free policy even if they put one into effect [11], and policies without cessation resources are thought to be minimally effective in promoting smoke-free living environments.

Given this hesitation, encouraging residents to voluntarily designate their homes as smoke-free represents one approach to introducing smoke-free living environments in the absence of a mandated policy. This strategy relies on education and voluntary behavioral change and therefore is aligned with PSH’s harm reduction framework. Smoke-free homes not only reduce exposure to SHS among non-smokers, but they also reduce smoking among smokers. In prior population-based studies, a smoke-free home was associated with reduced consumption, increased quit attempts [16, 17], reduced relapse to smoking, and increased effectiveness of smoking cessation aids [18]. Despite the fact that low-income households were less likely to have smoke-free homes, low-income smokers who had a smoke-free home had similar quit rates to higher-income smokers, suggesting that smoke-free homes could mitigate part of the income disparity in smoking cessation.

We previously conducted a pilot study to evaluate the efficacy of a multi-faceted intervention to increase voluntary adoption of smoke-free homes, i.e., a voluntary no-smoking rule in one’s home, with 100 formerly homeless adults in 15 PSH sites in the San Francisco Bay Area [19]. The intervention consisted of providing a step-by-step guide to residents on how to voluntarily adopt a smoke-free home and training PSH staff on how to refer residents to local cessation resources. At 6 months, 31% of residents adopted a voluntary smoke-free home compared to 12% at baseline.

### Study objectives

Here we describe a protocol for a cluster-randomized wait-list controlled trial to evaluate the efficacy of the same smoke-free home intervention on the primary outcome of voluntary adoption of a smoke-free home. This approach of encouraging the voluntary adoption of smoke-free homes offers a novel pathway to increase access to smoke-free living in housing where there is no mandated policy [20].

## Methods/design

### Study design

**Fig. 1:**
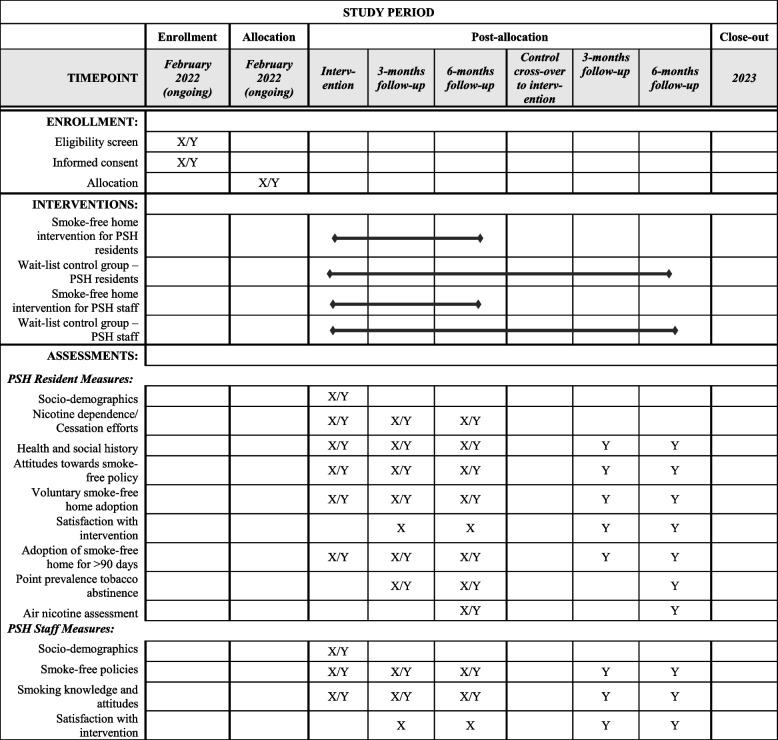
Randomization, recruitment, intervention delivery, and assessment schedule. Legend: X indicates measure or action for intervention group, Y indicates measure or action for wait-list control group

We will conduct a cluster-randomized, wait-list controlled trial [21, 22], where eligible PSH sites will be randomized into intervention and wait-list control groups. Those in the wait-list control group will receive usual care first and then cross over to the intervention arm after 6 months (Fig. 1).

### Intervention

The intervention, based on a train-the-trainer model, will include two parts: 1) study staff will deliver a one-hour counseling intervention to residents on how to adopt a smoke-free home, and 2) study staff will provide training to PSH staff on how to provide brief cessation counseling to residents and refer to local tobacco cessation services. The PI will train study staff and will assess fidelity by conducting observations of the counseling and training sessions and providing feedback to staff.

### Theoretical foundation

The individual intervention components are rooted in the social cognitive theory [23], which posits that behavior change takes place as a result of a dynamic and reciprocal interaction of the individual, behavior, and environment. We anticipated that by providing PSH residents knowledge and skills to adopt a smoke-free home, we would increase PSH residents’ engagement with the intervention. To scale up the intervention to multiple PSH sites and to lend rigor and structure to intervention development, we mapped intervention functions to the Behavior Change Wheel (BCW), a theoretical framework that describes barriers to and enablers of the behavior and offers nine possible intervention functions and seven possible policy options to affect behavior change [24–26]. The BCW domains and corresponding elements of the intervention are provided in Table 1. The BCW includes three layers [1]: the first uses the Capability, Opportunity, Motivation, Behavior (COM-B) [24, 25] model to highlight key barriers and potential enablers to voluntarily adopting a smoke-free home [2]; the second highlights five intervention options that cuts across individual, social, and organizational influences to address the barriers identified in the COM-B domains; and [3] the third offers three policy and programmatic options to disseminate the intervention [26]. Psychological capability reflects knowledge and awareness, and physical capability refers to skills necessary for behavior change. Opportunity comprises social and physical factors that make behavior change possible. Motivation includes the processes that direct behavior change, including emotional response, habitual processes, and cognitive processes.

**Table 1:** Mapping the smoke-free home intervention delivery to the Behavior Change Wheel

Barriers identified using the Capability, Opportunity, Motivation, Behavior (COM-B) model	Intervention functions (layer 2)	Policy targets (layer 3)
**PSH resident barriers and potential enablers** -Have decreased knowledge of SHS or thirdhand smoke *(capability)* -Lack skills to adopt a smoke-free home *(capability)* -Indoor cannabis use and other substance use reduces capability and motivation to smoke outdoors *(capability/motivation)* -^a^Kids/pets may encourage smoke-free home adoption *(motivation)* *- *Lack of safe designated smoking zones reduces opportunity for smoke-free living* (opportunity)* - Social norms of pervasive smoking reduces opportunity for smoke-free home *(opportunity)*	**PSH residents** -Education: Information on harms related to SHS and thirdhand smoke; combustible tobacco and e-cigarettes, and cannabis -Training: Step-by-step guide to adopt a smoke-free home -Restrictions: Voluntary smoke-free home adoption -Persuasion: Pledges; infographics on impact of SHS on kids and pets -Enablement: Referral to smoking cessation resources	**Environmental or social planning** -Voluntary adoption of smoke-free homes -Exploring potential downstream effect of other residents adopting smoke-free homes **Service provision** -Providing referrals to cessation programs
**PSH staff barriers** -Believe residents prefer to smoke indoors *(capability)* -Have no skills to refer residents to smoking cessation programs *(capability)* -Believe that smoking can allay a resident’s mental health symptoms *(capability)*	**PSH staff** -Training: To refer residents to smoking cessation programs -Education: Information from pilot data that residents are supportive of indoor smoke-free policies; debunk myths around nicotine allaying mental health symptoms	

^a^Potential enabler

Through our formative work and pilot study [11, 19], we identified that residents’ psychological and physical capability to adopt a smoke-free home was limited by lack of knowledge on the harms of SHS, and lack of skills on how to adopt a smoke-free home. PSH staff also reported a lack of knowledge needed to help residents implement smoke-free homes or access cessation resources. Opportunity was limited by lack of a safe designated smoking zone to smoke outdoors and on-site resources for smoking cessation. Awareness that SHS negatively impacted children and pets was a motivation for smoke-free home adoption; however, indoor co-use of substances decreased motivation to quit. Guided by the BCW framework, the resident intervention will include the following key components: education and skill building for PSH residents using a step-by-step guide on how to adopt a smoke-free home, training for PSH staff on how to provide brief cessation counseling, restrictions that include voluntary adoption of smoke-free homes, persuasion using imagery to encourage smoke-free home adoption, and enablement that includes provision of brief cessation counseling and referrals to cessation services. Study staff will also assist participants in calculating their personal financial costs related to tobacco use and encourage them to use the smoke-free home pledge to designate their home smoke-free. The intervention will be delivered using a culturally-tailored informational pamphlet.

The training for PSH staff includes webinar-style health education on tobacco use and cessation, nicotine addiction, and treatment of nicotine addiction using the 5As for smoking cessation or Ask, Advise or Refer model. Because PSH staff often play an essential role in linking residents to medical and mental health care, the training intervention emphasizes the importance of staff and empowers them to ask, advise, and refer [27] to smoking cessation services. The total time for the PSH staff training is 2 h.

### Randomization

We will randomize 20 PSH sites in the San Francisco Bay Area into the intervention and waitlist control arms using restricted randomization, a process which ensures an acceptable level of balance across groups at baseline [22]. We will match intervention and control groups by size of property, geographic location, and current smoking-related policies.

### Participants and setting

We aim to enroll 400 PSH residents, with 200 each in the intervention and control arms, with approximately 20 residents per site. Eligible PSH residents are those who are current smokers (smoked at least 100 cigarettes in their lifetime, and smoked daily or non-daily and at least five cigarettes per day in the past 7 days, verified by expired carbon monoxide [CO], ≥ 8 ppm [ppm]) [28], who smoke in their homes, expect to live in the PSH site for an at least 12 months, are 18 years or older, are English proficient and can provide informed consent.

We will also enroll PSH staff (*n* = 120) for a staff training intervention on how to provide brief cessation counseling. Staff are case managers, property management and administrative staff, nurses, or social workers who have a caseload of residents and ensure the day-to-day functioning of the site and coordinate services for residents. Eligible PSH staff are 18 years and older, are willing to participate in the two-hour training, and are able to provide informed consent.

The PSH settings included are clustered sites where housing and supportive services are delivered to all residents in a single, multi-unit building. These clustered sites can be small (< 30 housing units), medium (30–70 housing units), or large (70–130 housing units), and depending on the agency’s property portfolio, can include between one and 10 staff providing services at each site.

### Recruitment and enrollment

Study staff will recruit residents in person by posting flyers at study sites to provide information about the study and publicize the study through a “kick-off” community engagement event targeted to residents at the site. During the kick-off event organized at the PSH site community space, study staff will gather contact information from interested PSH residents and plan to enroll participants after eligibility assessment [29]. Study staff will use the teach-to-goal method to obtain informed consent from the participants [30].

### Control

PSH residents and staff in the wait-list control group will receive usual care, which in most PSH sites involves no interventions for smoke-free home adoption or referrals to smoking cessation resources.

### Study timeline and follow-up

The study duration for intervention group participants is 6 months. Wait-list control group participants will be offered the intervention after the intervention group participants within the same cluster receive their intervention and have the potential to stay enrolled for up to 1 year. Staff will collect data at baseline, 3 months, and 6 months post-intervention. The process is summarized in Fig. 1.

### Reimbursements

We will reimburse all resident participants with $20 gift cards for completing the baseline questionnaire, $15 for the three-month questionnaire, and $25 for the six-month questionnaire. We will pay participants $5 for monthly tracking visits between follow-ups and will pay those selected for random air nicotine monitoring at 6 months an additional $5 for keeping monitors in their home for 7 days. These amounts have been used in prior studies [19, 31–33]. We will reimburse PSH staff participants with $20 gift cards upon completion of the baseline questionnaire, $15 for the three-month, and $25 for the six-month questionnaire. We will offer snowball sampling to promote additional recruitment and offer enrolled participants an incentive of $10 for referring any eligible participants to this study, for up to three participants during the study duration.

### Retention

For each scheduled visit (i.e., three- and six-months follow-up), we will contact participants at least three times before they are marked as having a missed visit. We will offer monthly incentivized check-in visits (i.e. at two, four, and 5 months) between scheduled assessments that are in person or by telephone to facilitate retention for study follow-up visits. We will contact participants weekly for up to a month before being marked as having a missed visit.

### Data collection

We will build questionnaires on Research Electronic Data Capture (REDCap), a secure online data collection platform [34, 35]. Study staff will administer the questionnaires to resident participants, and staff participants will be asked to complete the questionnaires with assistance from study staff.

### Measures

#### PSH residents

##### Sociodemographic, residential history, and health status

At baseline, we will obtain information on age, sex (sex assigned at birth, current gender), race/ethnicity, education, monthly income (i.e., income from all sources), and health status [36]. For residential history, we will ask participants to provide the length of time they have lived in their current PSH, where they lived prior to current residence, and the number and length of episodes of homelessness in their lifetime [4].

##### Nicotine dependence and tobacco cessation history

At all three collection periods, we will assess Fagerstrom’s test for nicotine dependence [37]. We will collect cessation history including intention to quit, quit attempt (ever and in the past year), length of the last quit attempt, and use of cessation aids during the last quit attempt (e.g., medications, telephone quit line), and whether they have had encounters with housing staff around smoking cessation services.

##### Alternative tobacco and nicotine product use

At baseline we will obtain information on ever use, and at follow-ups we will obtain information on use in the past 30 days and number of days used of non-cigarette tobacco and nicotine products including electronic cigarettes, cigars, little cigars, smokeless tobacco, hookah/waterpipe, and blunts.

##### Chronic diseases

At baseline, we will ask participants whether they have received a diagnosis of liver, renal, or cardiovascular disease, hypertension, stroke, diabetes, cancer, arthritis, asthma, or chronic obstructive pulmonary disease [38].

##### Mental health

At baseline and follow-up, we will assess depression using the Center for Epidemiologic Studies Depression Scale [39], anxiety using a seven-item anxiety scale (Generalized Anxiety Disorder-7, GAD-7) [40], post-traumatic stress disorder using the Primary Care PTSD Screen for DSM-5 (PC-PTSD-5) [41], and urban stress using the Urban Life Stressors Scale [42]. We will also ask about history of diagnosis of schizophrenia or bipolar disorder.

##### Alcohol and substance use disorders

At all three collection periods, we will administer the Alcohol, Smoking and Substance Involvement Screening Test version 3.0 (WHO-ASSIST) [43–45]. In order to assess volume of alcohol consumed, we will administer the consumption questions from the Alcohol Use Disorders Identification Test (AUDIT-C) [46, 47]. To assess cannabis use, we will also administer the Cannabis Use Disorders Identification Test-Revised (CUDIT-R) [48].

##### Maltreatment, and subsistence needs

We will ask participants to report whether they had experienced difficulty meeting subsistence needs such as food, utilities, medications, healthcare, phone, clothing, childcare, or anything else in the past 12 months. Because racism and discrimination are central to experiences of homelessness and also related to tobacco use, we will ask participants to report these experiences using the Everyday Discrimination Scale (range 1[almost every day] to 6 [never]) [49].

##### Attitudes toward tobacco and cannabis smoke-free policy

At baseline and follow-up, we will ask participants to report whether they had experienced secondhand smoke exposure from tobacco and cannabis in the past month inside their apartment, in indoor shared areas like hallways or entryways, in outdoor areas like porches, patios or balconies, and other outdoor areas like the parking lot (never, hardly ever, a few times a month, a few times a week, or every day). We will ask them to report their level of agreement to a hypothetical smoke-free policy inclusive of tobacco and cannabis, including whether they would support such a policy, would try to cut down, would try to stop smoking, would continue to smoke in their apartment, or would try to move to another apartment because of the policy.

##### Voluntary smoke-free home adoption and smoke-free home rules

We will ask participants to report any rules around smoking in their home (there are no rules around smoking in my home, smoking is allowed anywhere, smoking is allowed some time or some places, smoking is not allowed anywhere in my home). We will ask participants to report whether they have attempted to establish a smoke-free home rule in the past 3 months, whether they have attempted to not smoke in their home in the past 3 months, and the number of days they were smoke-free in the past 3 months. These items will be used to define our primary outcome of self-reported voluntary smoke-free home adoption for 90 days or more at 6 months follow-up. We will ask participants to report their level of agreement to barriers to adopting a smoke-free home.

##### Satisfaction with intervention

At the follow-up, we will ask intervention participants to rate their satisfaction, usefulness, and quality of the materials using a Likert scale. In addition, we will ask about participants’ exposure to and use of smoking cessation materials provided by PSH staff.

#### PSH staff

##### Sociodemographic status

At baseline, we will ask staff to provide their age, sex assigned at birth and gender, race/ethnicity, role in the facility, and smoking history (if applicable).

##### Smoke-free policies

At all three data collection periods, we will ask staff about smoke-free policies, such as restrictions on indoor e-cigarette or smoked cannabis use, provision of smoking cessation resources, and enforcement of any smoke-free policies. We will ask about any experiences with challenges to policy enforcement or complaints about policies.

##### Smoking knowledge and attitudes

We will ask PSH staff to provide input on usefulness and satisfaction with the intervention and whether they have referred residents to smoking cessation programs. We will ask PSH staff to complete the Smoking Knowledge Attitudes Practices (SKAP) survey at baseline, 3 months, and 6 months post-intervention (score range 1–5, with a higher score indicating increased knowledge, beliefs, efficacy, and practices, and reduced barriers toward providing smoking cessation care) [19, 50, 51].

### Outcomes

#### PSH residents

The primary outcome is self-reported voluntary adoption of smoke-free homes for 90 days or more at six-months follow-up. We will place passive air nicotine monitoring [52, 53] devices in a random sample of intervention and wait-list control homes for a seven-day period at 6 months follow-up to verify self-reports of smoke-free home adoption. These monitors will be purchased from and analyzed via gas chromatography by the University of California, San Francisco (UCSF) Tobacco Biomarkers Core Laboratory. Study staff will obtain permission at the time of informed consent to place monitors in residents’ rooms for 7 days [52], within 1 week of six-month interview completion. Monitors will be placed at least 1 ft away from windows, corners of the room, and exits [52]. We will rely on the threshold of 0.9743 μg/m^3^ (sensitivity 69.5% and specific of 81.2%) for a home not being smoke-free [52]. Upon collection, samples will be stored in the UCSF Tobacco Biomarkers Core Laboratory prior to analysis. The secondary outcome is carbon monoxide-verified point prevalence tobacco abstinence at 6 months follow-up (expired carbon monoxide ≤5 ppm is defined as abstinence).

#### PSH staff

The primary outcome of interest for PSH staff is change in SKAP score from baseline to 6 months follow-up.

### Power analyses

This study is powered to detect medium-large effect sizes in the primary and secondary outcomes of interest for PSH residents (self-reported smoke-free home adoption and point prevalence of tobacco abstinence). This anticipated effect is consistent with estimates from the pilot study [19] and previous assessments of smoke-free home interventions which found over 40% change in the intervention group compared to a 25% change in the control group [52]. Power analyses assumed 80% power; two-tailed alpha of 0.05; *N* = 400 smoking residents recruited from 20 PSH units; 80% retention at 6 months; average six-month site cluster size of 16 residents; group comparisons of six-month binary outcomes describing adoption of a smoke-free home for ≥90 days and point prevalence abstinence from preliminary data, expected six-month control group outcome rates of 12 and 4% for smoke-free home adoption and point prevalence abstinence, respectively; from preliminary data, intra-site outcome correlations of 0.077 (*ρ*_SFH_) and zero (*ρ*_PPA_); and mixed logistic models including random intercepts for sites. Estimated design effects equaled 1 + (16–1)*ρ*_SFH_ = 2.16 and 1 + (16–1)*ρ*_PPA_ = 1.0, respectively.

For the primary outcome of voluntary smoke-free home adoption, the minimum detectable effect for the intervention group is 30.3% compared to 12% for the control group, which compares favorably with 31.3% obtained in our pilot study as well [19]. For the secondary outcome of point prevalence abstinence at 6 months, the minimum detectable effect in the intervention group is 12.6% compared to 4% in the control group, which compares favorably with 16.9% obtained in the pilot study [19].

### Statistical analysis

First, we will conduct descriptive analyses including means, proportions, variation, and confidence intervals of PSH resident socio-demographic variables including age, gender, and race/ethnicity. Randomization adequacy will be evaluated by considering whether baseline characteristics are independent of intervention group assignment, and attrition analysis will also be conducted. Missing data will be accommodated by multiple imputation [54–56]. If any key demographic or risk factors are imbalanced at baseline, propensity score-based adjustment will be included [57–59]. Then, we will analyze patterns of smoking cessation behaviors over time to determine the relationship between these practices and voluntary adoption of a smoke-free home. We will conduct primary analysis using intention-to-treat comparisons.

To account for the structure of these data where participants are measured repeatedly over time and are grouped by housing site, we will employ a mixed logistic model with random intercepts for sites and residents. The primary outcome is a binary indicator of smoke-free home adoption which, in an intention-to-treat analysis, will be regressed onto indicators of experimental groups, categorical time (baseline, 3 months, 6 months), and the group-by-time interaction. We will interpret and describe any significant intervention group main effect at follow-up and group-by-time interaction effect.

A secondary analysis will fit a mixed logistic model of the secondary outcome, point prevalence abstinence. Additional analyses will explore mediators (e.g., skills related to smoke-free home adoption, use of referrals to counseling) and moderators (e.g., children or pets at home, indoor cannabis use or other combustible tobacco and nicotine product use) of intervention effects on smoke-free home and point prevalence abstinence outcomes via mixed effects regression models. Replicability of within-group changes on primary and secondary outcomes will be tested by comparing baseline to six-month changes in the intervention group to corresponding six- to 12-month changes in the waitlist group.

Analyses of PSH staff data will include a pre-post training analysis of the smoking knowledge, attitudes, practices, efficacy, and barriers scale using mixed linear models. We will use mixed linear regression models to examine factors associated with change in each of the scales, clustering by site and staff participant ID, and adjusting for demographics, staff smoking status, encounters with residents on providing referrals for cessation, and staff role in the PSH site (e.g., case manager). In addition, we will examine unadjusted SKAP scores by PSH staff attendance of training, smoking status, PSH site and geographic location.

## Current status

At the time of the manuscript submission, the trial is actively enrolling participants. Recruitment began in February 2022, and we have rolled out the intervention in three blocks of six sites (i.e., 18 sites). Within each block, there are three intervention and three control sites. We have enrolled 129 participants to date and have completed 3 months follow-up data collection on 79 participants, and 6 months follow-up data collection on 20 participants. We plan to continue recruitment until we reach our target sample size, which we expect to reach by October 2023.

## Discussion

This protocol describes a voluntary approach to introducing smoke-free living environments in housing where there is no mandated policy or where a mandated policy may be difficult to enforce. A voluntary approach has the benefit of being self-enforced and has the potential to catalyze a normative shift in smoking behaviors. Early adopters of smoke-free homes could motivate a critical mass of residents in a building to voluntarily adopt a smoke-free home, potentially paving the pathway for a building to become smoke-free. The proposed study protocol offers a potentially useful and scalable approach for other types of multi-unit housing that are considering implementing smoke-free policies.

Federal legislation prohibiting smoking in public housing authority housing does not address other multi-unit living arrangements like housing choice voucher programs or PSH, leaving a gap in the implementation of policies in housing for people that are disproportionately impacted by tobacco use [60]. Intersectional populations including low-income, multi-generational families with elderly residents and children who belong to racial/ethnic minority groups are over-represented in subsidized housing and are vulnerable to the harmful effects of SHS and heightened morbidity and mortality from tobacco use [61–63]. Low-income multi-unit housing residents have a higher prevalence of tobacco use than the general population, due in part to tobacco industry targeting of these populations and increased tobacco retail density in low-income communities [64]. These structural and commercial determinants of tobacco use highlight an urgent need for comprehensive smoke-free policies in all forms of multi-unit housing [64].

Current implementation efforts of smoke-free policies in public housing authority housing present important lessons for enforcement. A pre- and post-study that evaluated the effects of the 2018 federal ban on smoking in public housing on air nicotine concentration levels in New York City public housing, found no significant difference in nicotine levels between living areas where the policy was in effect to areas where there was no policy [65]. In a mixed methods evaluation of the federal smoke-free public housing policy in New York City, residents saw limited enforcement of the ban in the year following its implementation [66]. Moreover, qualitative findings suggested that there would be resident and staff resistance to a mandated smoke-free policy [66].

In the context of these challenges, smoke-free home initiatives that include resident endorsement and provision of a designated smoking zone provide a promising alternative because they offer a self-enforced approach to having an indoor smoke-free policy [67]. A voluntary, self-enforced approach could facilitate transition to a building-wide smoke-free policy or increase impact of a smoke-free policy that is in place. Further, smoke-free home interventions that are tied to proven smoking cessation services (such as behavioral counseling and pharmacotherapy) are poised to promote a supportive environment where people are able to quit smoking [20]. Results from our pilot study showed that 17% of participants reported carbon-monoxide verified abstinence at 6 month follow-up, and self-reporting a smoke-free home was associated with abstinence [19].

Housing is an important social determinant of health. People who have experienced homelessness have also faced lifelong trauma and chronic housing instability. Access to affordable housing including supportive housing and services is the foundation for health [68]. When those managing affordable housing demonstrate commitment to reducing environmental exposures like tobacco and the accompanying risk of tobacco-related morbidity and mortality, there is a greater opportunity for recovery. By promoting smoke-free homes and access to cessation services, we capitalize on the opportunity for housing to be the foundation of health and recovery.

## Data Availability

No data were analyzed as a part of this protocol paper. Dr. Vijayaraghavan will have full access to all the data collected during the cluster randomized controlled trial and will take responsibility for the integrity of the data and the accuracy of the data analysis. Data will be made available only upon request and in accordance with an established data sharing agreement.

## Abbreviations

PSH: Permanent supportive housing
SFH: Smoke-free home
SHS: Second-hand smoke
